# Remembered and Forgotten: The Nineteenth-Century Flemish and Dutch Famine in Cultural Memory

**DOI:** 10.1080/03096564.2025.2441531

**Published:** 2024-12-25

**Authors:** Lotte Jensen

**Affiliations:** Faculty of Arts, Radboud University Nijmegen, Nijmegen, The Netherlands

**Keywords:** famine, cultural memory, Flemish movement, Hendrik Conscience, Dutch potato crisis

## Abstract

In the years 1845-48 Belgium and the Netherlands suffered from famine as a result from potato blight. This article explores the question why this famine became a building block of Flemish identity leading to its inclusion in the Flemish canon, while the Dutch potato crisis still largeley remains forgotten. It is argued that the famine in Flanders was intrinsically linked with the nineteenth-century Flemish movement, thereby contributing to the self-image of ‘’poor Flanders’’. By contrast, the emergence of Dutch identity goes further back in history and is mainly connected to prototypical disasters, such as floods. Famine did not suit this storyline and the ‘’superior’’ ability of the Dutch to manage the water. This article also stresses the need for transnational approaches in the study of famine memories. A comparative approach makes it possible to understand why the same type of traumatic event was forgotten relatively quickly in one case, and became part of the collective memory in the other.

The Flemish people moan, like the Irish,
About a punishment they did not deserve
They both find no mercy
But in the abyss of the grave.^1^

With these verses, written in April 1847, the Belgian poet Theodoor van Ryswyck lamented the hopeless situation in which many people in Flanders found themselves. In ‘Vlaenderens Ramp. Politiek Referein’ (‘The Disaster of Flanders, Political Refrain’) he sketched the bitter reality of thousands of people dying of hunger, waiting in vain for help. He compared their situation with that of the Irish, among whom famine also raged mercilessly.

In the years 1845–49 several European countries experienced failed harvests due to the potato blight *phytophthora infestans*.^2^ In Ireland, the Scottish Highlands, Belgium, the Netherlands and Prussia this caused wide-scale famine that killed tens of thousands.^3^ In Ireland, it is estimated that over one million people died from hunger or hunger-related diseases, which resulted in a 12% decrease of the country’s population.^4^ Many more fled the country, seeking refuge in the United States and Canada. The mortality rate was not that high in Belgium and the Netherlands, but the consequences of the failed potato crops were still very dramatic: in Belgium, total excess mortalities in 1846–1848 may have been as much as 40,000–50,000 deaths.^5^ The majority of deaths occurred in East- and West Flanders, that suffered heavily from the decline of the traditional linen industry.^6^ In the Netherlands in the years 1846–47 there was an excess in mortality of 22% and 32% respectively, which may roughly equate to 50,000 deaths as well. The exact number of deaths, however, is impossible to ascertain because many factors came into play. The combination of wheat harvests and very cold winters, also led to high food prices and increased poverty amongst the Dutch population in general. During the years 1848–49 outbreaks of cholera worsened the situation.^7^

Of these famines, the Irish potato famine has been studied by far the most extensively, not in the least because the food crisis led to mass emigration.^8^ The 150^th^ anniversary in the mid-1990s in particular generated commemorative attention across Ireland and the nations of its diaspora.^9^ The many public commemorations and cultural media, such as monuments, literary texts, paintings, children’s books and exhibitions, have contributed to making this event a benchmark of Irish national identity.^10^ The famines in the Low Countries have been investigated less extensively, and primarily through a socioeconomic lens.^11^ It is, however, also worthwhile studying the memorial cultures of the famines in this area from a comparative perspective. Such approach focuses on ‘heritages of famines’ and tries to explain why and how certain food crises are remembered, while others are relatively quickly forgotten.^12^

The memorializations of the Belgian and Dutch famines followed a completely different course. The potato crisis in the Low Countries has long been part of what Aleida Assmann calls the ‘passive dimension of cultural memory’: it is ‘stored and potentially available’ in the archives, but not interpreted or made accessible in the public sphere.^13^ Comparing Flemish and Dutch cultural memory of the potato crisis, however, reveals some interesting differences. In the Netherlands, the potato crisis was forgotten relatively quickly. When people nowadays refer to famine in the Netherlands, they usually think of the Dutch Hongerwinter (1944–1945), which occurred during the Second World War.^14^ In Flanders, on the other hand, there have been many more cultural and literary responses, which resonated during the nineteenth and twentieth centuries. At this moment, we are witnessing a remarkable process of retrieval: in 2023, the potato crisis was selected by a committee of historical experts as one of the sixty entries in the ‘Canon van Vlaanderen’ (Canon of Flanders), making it one of the key events in Flemish history.^15^

This article explores the question why and how the famine in Belgium became a building block of Flemish identity leading to its inclusion in the Flemish canon, while the Dutch potato famine still largely remains forgotten among the broader public. Firstly, I will discuss the Flemish cultural responses to the famine in the past and the present, subsequently shifting the focus to the Dutch remembrance of this famine down the years. By cultural responses is meant: articles in newspapers, brochures, essays, poetry, novels, stories, musical pieces, and so on. For Flanders, only some of the sources are discussed to give a first impression of how the crisis was responded to, for the Netherlands it is almost exhaustive. That alone shows that there was a significant difference in both areas. The third section will discuss the differences between the Flemish and Dutch dealings with the famine and proposes several explanations. I will argue that the famine in Flanders was intrinsically linked with the nineteenth-century Flemish movement, thereby contributing to the ‘auto-image’ of ‘poor Flanders’.^16^ By contrast, the emergence of Dutch identity goes further back in history and is mainly (but not exclusively) connected to other prototypical disasters, such as floods. This article also stresses the need for transnational approaches in the study of famine memories: a comparative approach makes it possible to understand why the same type of traumatic event was forgotten relatively quickly in one case, and became part of the collective memory in the other.^17^

## Cultural Remembrance of the Flemish Famine

In June 1845 the potato blight first appeared in Kortrijk, after which it quickly spread to the rest of the country. Although the province of Wallonia was also affected, the consequences for Flanders were greatest: almost 90% of the harvest failed, which led to an acute food crisis with famine, diseases and death.^18^ The Flemish countryside, in particular the flax-producing regions, were hit hardest. Epidemics of typhus and cholera, in combination with high unemployment, plunged Flanders into a deep crisis.^19^ In several villages and cities this resulted in social protests and food riots.^20^

The relatively high mortality in Flanders explains why the food crisis was quickly perceived as a predominantly Flemish problem. Contemporaries described this as ‘le mal des Flandres’ or ‘la misère des Flandres’ (the misery of Flanders).^21^ By the end of the nineteenth century, the expression ‘Poor Flanders’ (‘Arm Vlaanderen’) was a commonplace, sometimes referring to the particular crisis of the 1840s, but more often to the poor social conditions of the rural population and factory workers in the cities in general.^22^

In the years 1845–1848 many Flemish authors drew attention to the suffering of the Flemish people. They combined their portrayal of the starving population with a plea for the emancipation of Flanders. The distress thus became instrumentalized for spreading patriotic feelings and promoting the ‘Flemish cause’. Periodicals and magazines played an important role in this.^23^ A case in point is the periodical *De Vlaemsche Stem. Tijdschrift ter bevordering van de Vlaemsche zaek* (1846–1853) which was launched by Jaak van de Velde, Pieter Ecrevisse and Domien Sleeckx. The editors criticized the dominance of French media in Flanders and promoted Flemish literature and culture as a spearhead of the Flemish movement. The periodical contained a mixture of essays, prose fiction and poetry, and attracted a broad range of authors, including Prudens van Duyse, Johan M. Dautzenberg and Constant Duvillers.^24^

One of the recurring topics in *De Vlaemsche Stem* was the famine in Flanders. The periodical regularly published poetry about suffering families, in particular mothers, children and widows. Edmond Ronse, an archivist and poet from Veurne, wrote about two brothers, who leave their mother behind to search for food. One of the brothers dies, succumbing to hunger and cold. In the final stanza’s he longs for his mother. Then his voice weakens, he sinks to the ground and is covered by the snow, which serves as his shroud.^25^ The poet O. J. Eekma, living in Zaltbommel in the Netherlands, expressed his sympathy with the suffering people in Flanders by portraying two sisters, one extremely poor and the other rich. The wealthy sister refuses to help her starving relatives, and is visited by the ghost of her mother in her dreams. She gets her punishment, dying because of her cold-heartedness. The moral message was to help the needy.^26^

One of the most outspoken contributions in *De Vlaemsche Stem* came from the East Flemish pastor Constant Duvillers. He published two rhymed ‘Pleas for the poor’ and asked everybody who could spare a penny to help them. He tried to evoke sympathy by sketching the despair of a helpless mother who could not feed her children and the emaciated people who fled to remote places in search for food. He uttered strong criticism against rich people who refused to help the needy. It was inhumane to reject the poor: ‘going hungry is so bitter!/And begging is so painful!’, he repeatedly stated.^27^ His ‘Second plea for the poor’ was also included in an anthology of Flemish poetry, containing a selection of ‘the best authors’: *Vlaemse bloemkorf, keus uit stukken van de beste schryvers* (1853).^28^

The contributions in *De Vlaemsche Stem* were often centred around the same themes: children searching for food, helpless mother figures and appealing to the conscience of the wealthy to be merciful. Such images also circulated in Irish famine literature of that time.^29^ The attention in this periodical intensified in reaction to a speech by the physician Joseph-Désiré Sigart in the Belgian parliament on 12 December 1846, in which he suggested that the poverty and hunger in the Flemish countryside was caused by the inferior character of its inhabitants.^30^ Sleeckx, one of the editors of *De Vlaemsche Stem*, was one of the people who showed his profound disapproval of such rhetoric.^31^ In his essay ‘Flemish self-sacrifice’ he meticulously sketched the pitiful circumstances in which the Flemish people lived. His purpose was to sketch the ‘real character’ of the inhabitants of Flanders to refute those people who meant to drive a wedge between Wallonia and Flanders by portraying the inhabitants of Flanders as degenerate bastard children. On the contrary, he wrote, it was in times of disaster one could best measure the degree of enlightenment and civilization of a people. The cruel famine made two exalted virtues of the Flemish people visible: the exemplary charity of the wealthy and the angelic resignation of those in need.^32^

This piece by Sleeckx makes clear that the food crisis and Flemish community-building were intrinsically linked. Being able to express the noblest virtues in times of distress became part of the positive self-image of the Flemish people. Pointing out the special characteristics of the Flemish people went hand in hand with a call for the emancipation of the region with regard to the language (Dutch instead of French) and social justice.^33^ A wave of social poetry flooded the market, sometimes explicitly thematizing the famine. Duvillers, for example, published a collection of folk songs (*Volksliedjes*, 1846), containing pieces about a wandering orphan, old beggar, and a suffering factory worker. All songs were set in Flanders and some of them contained typically Flemish expressions or dialect. *De Vlaemsche stem* exuberantly praised the songbook, in particular the number about the beggar with these telling lines: ‘I now sing out of dire need / I sing for my bread’.^34^

Many other well-known Flemish authors, including Theodoor van Ryswyck, Jan van Beers, Pieter Frans van Kerckhoven, August Snieders, Eugeen Zetterman, Maria van Ackere-Doolaeghe and Hendrik Conscience, also wrote about the famine and related issues such as hunger, poverty and illness. Van Beers, for example, wrote a sensitive poem ‘At the cradle of a child of a poor’, in which hunger forces a widow to go begging – in vain.^35^ Van Kerckhoven also published several poems about families in despair. In his poem ‘Vrouw Lisa’ a young mother is found dead with her children on her bed by her neighbour hours later.^36^ Poems like these often followed the same template: mothers are desperately trying to find food for their starving and freezing children. In the end death comes as a salvation: often the protagonists died with a smile on their faces. This refers to the fact that they go to heaven, a subtle but clear message of religious consolation.

The work of Hendrik Conscience, best known for his historical novel *De Leeuw van Vlaanderen* (1838), deserves special mention. On 7 March 1847 he contributed a speech about the victims of the famine in Flanders to a music festival in Antwerp. Flanders, he reminded the audience, was once a symbol of wealth, industry, thriving culture and heroism, but had now become the equivalent of ‘a prayer, a plaint, which rose from the immeasurable grave, a death scream that perishes on the lips of the dying.’^37^ The country had become ‘the realm of famine’.^38^ Conscience gave a chilling sketch of this realm of death: a textile worker was lying on his broken loom, with the dead body of one son next to him and the other crying at his knees. The mother presses her baby to the breast and wets its lips with her tears. He ended the heartbreaking sceneries by calling upon his fellow countrymen to donate food, clothing and money. Not only human lives were at stake, but also the existence of Flanders: ‘And maybe – the ghost of the future is calling at my ear ‒ the beautiful Flanders will lift the head from the grave, and shine in the heavens of nations as the star of industry and people’s power.’^39^

At the music festival, organized by the rhetoric chamber De Olyftak, with the help of the ‘ladies of charity’ and the ‘Nederduitsche zangmaatschappy’, several other authors made an appearance, including J. A. Delaet, Van Kerckhoven, Van Ryswyk, Beers and L. Vleeschouwer. According to the reporter of the newspaper *Het Handelsblad*, several members of the city council and notables were present at this event. Besides helping the poor, it was also a celebration of the ‘mother tongue’ and a celebration of Flemish history.^40^ Pieces of music were dedicated to medieval heroes such as Jan van Breydel and Jacob van Artevelde, iconic figures of the Flemish movement. Propagating solidarity with those in need and spreading patriotic feelings thus went hand in hand.

In addition to this speech, Conscience also wrote fictional stories about poverty and hunger. In 1872, he published a short novel, entitled *Een goed hart* (A good heart). The book, which also contained two illustrations by Edward Dujardin, was set in January 1847 and tells the story of the sixteen-year-old Victor Leemans, who wants to help the starving people in the ‘realm of famine’.^41^ When he and his friend enter a tavern, remarkably enough, one of the patrons is just reading aloud fragments of the speech Conscience gave at the music festival in Antwerp. Victor decides to take a starving mother and her two children into his home in Brussels, inspired by what others say: ‘Curing the ill, consoling the suffering, what is more beautiful and noble on earth?’.^42^ Years later he meets again the girl whom he saved from starvation at the theatre. She has become a famous singer and they get married. The moral message of the novel is that one must start saving a few people if one wants to save thousands; in other words: those who can, should offer help.

It should be noted that this story appeared more than twenty years after the food crisis, which is an indication that the famine remained present in the repertoire of authors and their readers. The crisis was also mentioned in a volume about the history of Belgium by Dautzenberg and Prudens van Duyse, which was published in 1856 and reprinted several times. In their entry on the famine of 1044 they wrote that Flanders was also hit very hard by famine in 1846: trade came to a standstill and the potato harvest failed.^43^ As terrible as that was, the disaster was greater in 1044. The circulation of prose and poetry about the Flemish famine was also ensured by the many reprints and collected volumes of Flemish poets that appeared in the decades after the famine. The remembrance of this dark period was, for example, commemorated by the poetess Maria van Ackere-Doolaeghe, who published a commemorative poem about the ‘potato plague of 1845’ in 1869. She first paints an idyllic picture of the fertile fields of Flanders, and then switches to the year when the plague entered: the sky turns black, Europe is startled, while people kneel before altars liking for aid and mercy from God. Thousands of families die where distress and hunger prevail. Even charity is not enough to bring relief. Trying to resist God’s will makes no sense, because His ways are mysterious, the poetess concludes. Courage to endure the cruel circumstances is the only thing that remains: ‘The Will that demands submission,/will comfort, heal after the flogging;/Courage, mortal, courage, to endure the fate’.^44^

The famine and social deprivation of the Flemish countryside thus became a lever in the development of Flemish self-awareness.^45^ On the one hand, authors underscored at the necessity of solidarity amongst Flemish citizens, while, on the other hand the distress nourished feelings of deprivation compared to Wallonia. Promoting the Dutch language and better social conditions became part of the same discourse, and paved the way for the Flemish movement in the second half of the nineteenth century and the twentieth century. Studying the rise of this movement therefore implies acknowledging the impact of the famine years, which also becomes clear from the attention to this period in the work of, amongst others, Lode Wils, Karel van Isacker, Gita Deneckere, Maarten Vanginderachter and Kevin Absallis.^46^ The cultural memory also stayed alive through fictional works, like stories and novels. The work of Conscience was reprinted many times and made publicly available online, and André Demedts wrote a historical novel about the Flemish famine: *Geluk voor iedereen* (1982).^47^ The topic was also included in a series of historical stories for teenagers. In *Hongersnood in Vlaanderen* (1954, reprinted in 1975) Emiel van Hemeldonck told the story of a poor family, who try to survive by sending the son to work in one of the factories in Ghent. One day his sister visits him to tell that their mother and sister have died due to typhus. The food riots in several cities are also described in detail.^48^

The recent inclusion in the World History of Flanders (*Wereldgeschiedenis van Vlaanderen*, 2018) and the Canon of Flanders (*Canon van Vlaanderen*, 2023) is illustrative for the recent revival of interest in the famine. Knowledge about the potato crisis and its causes are also transmitted to wider audiences through educational websites of the Centre of Agricultural History (Centrum Agrarische Geschiedenis).^49^ In conclusion: a wide variety of literary contributions, historiographical publications and educational websites have contributed to the lasting memory of the Flemish famine.

## Cultural Remembrance of the Dutch Potato Crisis

As in Flanders, the Dutch population was hit hard by the potato blight. A combination of failed wheat and potato harvests and very cold winters led to high food prices and increased poverty. In several Dutch cities, including Delft, Leeuwarden, Harlingen and Groningen, food riots broke out. Five people died in the food riots in Groningen.^50^

Journalists responded to the crisis by writing articles in the newspapers about taxes, food prices, social unrest, and charitable activities. Although the number of literary authors who wrote about the famine was much lower than in Flanders, these cultural responses nevertheless deserve our attention: some of the best-known authors wrote pieces about the food crisis. It should, however, be kept in mind, that the following presentation is more or less exhaustive, which indicates the modest attention paid to this topic amongst Dutch authors.

On the occasion of Christmas Eve 1845, the poet and clergyman Nicolaas Beets wrote a song, entitled *Troost der armen* (Consolation of the poor). In it he described the dramatic consequences of the potato blight for the Netherlands. Except a handful of cabbage, there was hardly anything left to feed the children. The main message, however, was that the poor should keep faith and trust in Jesus and God. True wealth lay in the sense of humility, Beets concludes.^51^ Beets’s song was sold for five cents each, or twenty-five copies for one guilder, and the proceeds benefited the poor.^52^ It was performed at a benefit concert for the poor in ‘s-Hertogenbosch in 1848, reprinted in Beets’s complete works and set to music in 1872 by J. J. van Krieken. This two-part composition could be used at schools.

Another prolific author who wrote in the benefit of the poor, was Hendrik Tollens. He became famous for his patriotic verse and wrote a new national anthem in 1817. He also wrote charity pieces in the wake of disasters, and considered it his societal duty to put his pen in service of the less fortunate. In 1848 he published a ‘Begging Letter in the Winter’ (*Een bedelbrief in den Winter*) to raise money for the poor people in his village of Rijswijk. He had already written this in the severe winter of 1844–45, but decided to have it printed, when the crisis worsened as a result of the potato blight and bad weather conditions. With his poem, in which he encouraged the middle and upper classes to donate goods and money, he raised 600 guilders. Thousands of copies were spread in the country, for which Tollens received praise by friends and relatives.^53^ The poem was reprinted in Tollens’s complete works.

A third well-known writer at that time was Cornelis van Koetsveld. He attained fame with *Schetsen uit de Pastorij te Mastland* (1843), a collection of moral sketches set in a small village, which were based upon his own experiences as a Protestant vicar in Westmaas. In 1847 he published a short story in *De Nederlandsche Volks-almanak* about an emigrant, ‘Klaas de Landverhuizer’, an anti-emigration text. The story is about a man who decides to leave his village, sell his home and grocery store, and move to America with his family. The departure plans meet disappointment: the passage fares are too high and the family has to return home. The religious message which Van Koetsveld wants to communicate to his audience is clear: people should rely on God, and remain faithful and patient in times of distress. One day, God will reward pious behaviour and bring back potatoes and bread to their lives.^54^

Probably the most widely read response came from Ottho Gerhard Heldring, who was also a vicar as well. In 1827 he was appointed in Hemmen, a small village in the province of Guelderland. There, he was confronted with the immense poverty of the rural population. He considered it his calling to improve the conditions of the poor. According to him, the solution lay in labour and education, on the one hand, and fighting immoral practices such as alcoholism and prostitution, on the other. His philanthropic writings were spread and read across the nation, including his brochure about the potato crisis: *Wat te denken van en wat te doen in den aardappelennood?* (What to think of and what to do during the potato crisis, 1845). He offered a detailed descriptions of the horrible conditions in which many citizens found themselves in, deprived of earnings and food. He also published about the terrible sights of starving people in the national newspaper *Algemeen Handelsblad*, and described how approximately four hundred beggars had visited his home during the three days before Christmas in 1845.^55^ His reports led to spontaneous donations and collection actions, although never enough to help everyone in need. Heldring concluded that the potato, in a way, was like the tower of Babel: people had placed all their hope on one vegetable, but the tower collapsed: ‘This time the potato was the tower of Babel, which the poor had their eye on, the bread-tree by which they lived’.^56^

The potato crisis also intensified the debate about poor relief and the question whether the central government had a role to play in this. The minister of internal affairs, Johan Rudolph Thorbecke submitted a bill regulating poor relief, but it was rejected. In 1854 a bill passed in which it was decided that poor relief was the task of deaconries and private institutions and initiatives. After that, the public interest in this topic quickly waned. The famine was also quickly forgotten. Except for the reprinted song by Beets hardly any references to these catastrophes can be found in the newspapers or literary works. New disasters drew the attention of the audience, such as the floods of 1855 and 1861, and the third large outbreak of cholera in 1866.^57^

That situation has remained largely unchanged to this day: there are no monuments, films, exhibitions, websites or documentaries in the Netherlands devoted to this famine. One exception, however, must be mentioned. In 2000 Simone van der Vlugt, a best-selling author known for her historical novels for both adults and adolescents, published *Zwarte Sneeuw*. This adult novel is set in Limburg in 1845. Due to failed potato harvests, a family suffers from hunger and poverty.^58^ They have to sell their farm and move to a larger town in order to survive. Then the novel shifts to the harsh working conditions of the miners in the region. Although hunger is an important theme, there is relatively little attention for physical suffering, starvation and death ‒ on the contrary. The focus lies on a scary adventure in the mines, the joyful moments when there is special food to share, and the novel has a happy ending. Unlike the Flemish literary works, the famine is embedded in a longer time span, and not highlighted as a special episode in Dutch history. The book, notably, won Flemish children’s literature award, ‘De Kleine Cervantes’ and was also translated into German by Eva Grambow, as *Emma: die Zeit des schwarzen Schnees* (2001).

We are currently witnessing processes of retrieval through which this famine is re-entering public consciousness, but it is difficult to predict how durable this memory will be.^59^ The attention is dwarfed compared with the national commemoration of the flood of 1953 and the upcoming events to commemorate the flood of 1825.

## Explaining the Differences in Cultural Remembrance

The contemporary responses to the famine in Flanders and the Netherlands instantly make clear the quantitative difference. In Flanders many more authors wrote about the horrific conditions people were living in, and the need to help them; in the Netherlands the newspapers did write about this topic including the food riots, but only a handful of authors, most of them vicars, addressed this issue. In Flanders, the public attention for the potato crisis continued well into the twentieth and twenty-first centuries, leading to the inclusion of this event in the Canon of Flanders, while the Dutch famine fell into oblivion almost immediately: in contrast with Flanders hardly any cultural responses or commemorative acts can be witnessed after the crisis.

How can this difference in the memorial cultures in the neighbouring countries, who shared their language and had been a single political unity during the years 1815–1830, be explained? In an overview article, Camilla Orjuela, professor of Peace and Development Research, discusses seven ways in which famine memorialization is impeded or made possible: narrative challenges, shame and culpability, competing traumas, breaking with the past, building the nation, instrumentalising the past in domestic and international politics and, finally, memory activism and local initiatives.^60^ Narrative challenges are characteristic of this type of disaster in general, a point that art historian Emily Mark-FitzGerald also stresses in her work: the ‘lack of central characters, linear narrative, heroic episodes or key dates’ make famine less suitable for commemoration compared to other disasters such as earthquakes and floods.^61^ Both the Flemish and Dutch authors may have struggled with this issue. Shame and culpability on the collective level can also be an obstacle to famine memorialization: the lack of food may be perceived as a shameful period in a nation’s or community’s history. However, this obstacle applied to both Flemish and Dutch authors and cannot explain the difference in attention. The issue of shame and culpability – the idea that a community wishes to forget this shameful episode of the past does not seem relevant either in this particular case. The other themes, however, may have played a part to a greater or lesser extent.

The issue of competing traumas, for example, has definitely impacted the memorialization of the potato crisis in the Netherlands. In the nineteenth century several other great catastrophes took place, in particular some major floods. The floods of 1809, 1820, 1825, 1855 and 1861 left many traces in Dutch cultural history, including writings, songs, memorial objects, and monuments. Dutch remembrance culture was, and still is, profoundly influenced by the so-called ‘battle against the water’, which continues to this day.^62^ Whenever a new flood or high water threatens the safety of Dutch citizens, a vast cultural repertoire is tapped into. These iconographies also shape a sense of pride and are intrinsically linked to community building, at the regional and the national levels. Local communities and the national government participate in countless memorial activities of floods from the past.^63^ The remembrance of the Sea Flood of 1953 has become part of the Canon of the Netherlands, and plays a constitutive part in telling the story of collective suffering and recovering ‒ in other words ‒ of Dutch solidarity and resilience. Famine did not suit this storyline of the emergence of Dutch identity and the ‘superior’ ability of the Dutch to manage the water. Worse even: hunger and poverty were portrayed as something shameful by the vicar Heldring, who considered it a flaw of society that so many people were starving to death. ‘Who am I’, he wrote, ‘that I as a rich man have plenty at the table, while the poor man lies there in his misery full of sorrow’?^64^

The opposite is the case in Flanders, as the overview of cultural responses demonstrates. There, the famine was, from the outset, framed as an experience of shared suffering, rather than as divisive or shameful. It became part of a narrative of Flemish deprivation compared to Wallonia, and nourished the emancipation struggle of Flanders in economic and cultural respects. It coincided with the rise of the Flemish movement and became an integral part of the collective story of this region: one of suffering and struggling for better conditions. Although historians of the Flemish movement have acknowledged the huge impact of the famine, the connection with the famine and the representation of this crisis deserves further analysis: was it also instrumentalized in internal political conflicts, and how does it relate to views on the Walloon side? Is the renewed attention perhaps also influenced by the heightened interest in the commemoration of famine in Ireland?

Another issue that deserves investigation is the renewed attention for the food crisis in recent years. The establishment of a Canon of Flanders went hand in hand with intense public debates, in which historians also took part. Some argued that history was used as a political instrument to spread Flemish populism, while others considered this a great opportunity to spread historical knowledge to broader audiences including school children.^65^ Critics questioned why a Canon of Flanders had been established instead of a Canon of Belgium. These discussions were reinforced by the fact that more or less at the same time, a television series was launched, The Story of Flanders (*Het verhaal van Vlaanderen*), presented by Tom Waes. In this series, which revolved around a collective ‘we’, the potato crisis was notably absent – a missed opportunity according to some of the critics.^66^ Paradoxically enough, noticing this absence underlines the canonical status of the Flemish famine; in the Netherlands nobody would have expected the famine to be part of the Story of the Netherlands (*Het Verhaal van Nederland*), which was broadcast in 2022. References to the struggle against the water, and living in a vulnerable delta, however, were expected to be included, and are also very prominent present in the Canon of the Netherlands.

## Conclusion

The memorialization of the nineteenth-century famines in Flanders and the Netherlands, caused by the potato blight, show a different course. In Flanders virtually every leading author wrote about the consequences for their region, while the number of Dutch cultural and literary responses was limited to a handful. The biggest difference concerns the impact of this troubled past. In Flanders a direct line can be traced from this particular episode of the region’s history to the expression ‘the misery of Flanders’, which became commonplace to describe the poverty of the rural population and the poor working conditions of factory workers in the cities. It also became connected to the idea that the poor Flemish people were deliberately oppressed by the Walloons and French-speaking elite. This idea already circulated during the famine and lived on during the nineteenth century in the Flemish movement. In the twentieth and twenty-first centuries historians investigated this topic in relation to the Flemish movement, which also brought it back in the public sphere via larger memory projects such as the Canon of Flanders.

In the second half of the twentieth century the Flemish economy would surpass that of Wallonia, and nowadays the balance is fully shifted. Flanders is considered to be the rich half of Belgium. The potato crisis still serves, even with renewed visibility in the most recent years, as a decisive moment in Flemish history. It tells the story of the long-term deprivation, oppression and finally resurrection of a region that was part of a divided nation, and was (and still is) in search of its own identity. The comparison with the Netherlands, where this catastrophe also led to a serious food crisis and uprisings, could not be greater. Embracing its own prototypical disaster to magnify the differences with the Netherlands, from which Belgium seceded in 1830, may also have played a role in the background. The transnational perspective chosen here once again clarifies how disasters are instrumentalised very differently across borders, in the process of community building.

## Notes

1. This article was supported by Netherlandish Organization for Scientific Research grant number, NWA 160.18.197. I would like to thank Marguérite Corporaal and Chris Cusack for their comments, and Kevin Absillis and Gita Deneckere for their reading suggestions. “De Vlamingen, als de Ieren kermen,/Om een nog nooit verdiende straf;/Zy vinden beiden geen erbarmen/Dan in de afgrond van het graf.” Van Ryswyck, *Volledige werken*, vol. 3, 272.

2. For a comparative overview, see Vanhaute, Paping and Ó Gráda, “The subsistence crisis of 1845–1850”.

3. Paping Vanhaute, and Ó Gráda, “The subsistence crisis of 1845–1850,” 37.

4. Mokyr and Ó Gráda, “Famine Disease,” 37.

5. See 3 note above,” 26.

6. See *De Canon van Vlaanderen*, 178.

7. Paping and Tassenaar, “The Consequences of Potato Disease,” 176.

8. By, amongst others, Ó Gradá, *Ireland before and after the Famine*; Gray, *Land and Politics;* Donnelly, *The Great Irish Potato Famine* and Ó Murchadha, *The Great Famine*.

9. Mark-FitzGerald, *Commemorating the Famine.*

10. Mark-FitzGerald, *Commemorating the Famine*; Corporaal, *Relocated Memories*.

11. For the Netherlands, see Paping and Tassenaar, “The Consequences” and Curtis et al. “Low Countries. For Belgium, see: Van Isacker, Mijn land in de kering, Deneckere, “Sire, het volk mort” and Van Ginderachter, “Arm Vlaanderen”.

12. The project “Heritages of Hunger” dealt with the cultural memory of 19^th^ and 20^th^ century famines in a selection of European countries, see: Corporaal and De Zwarte (eds.), *Famines and the Making of Heritage*.

13. Assmann, “Canon and the Archive,” 36. Cf. Corporaal and Jensen, “Famine and ‘hongersnood’ as transnational memory”.

14. De Zwarte, *The Hunger Winter*. On the cultural memory of the “Hunger Winter,” see De Zwarte and Jensen, “Relative Absence”.

15. *De Canon van Vlaanderen*, nr. 32 “De aardappelcrisis,” 178–181. Also online accessible: https://www.canonvanvlaanderen.be/events/de-aardappelcrisis/.

16. This concept is used within Imagology, the critical study of national stereotypes: Leerssen, *Echoes and Images*, 129.

17. This need for transnational approaches in disaster studies is also emphasized by Corporaal and Jensen, “But most brothers in misfortune” and De Zwarte and Janssen, “Recalling the Hunger Winter”.

18. On the causes and spread of the potato blight, see Bruneel, Beeckaert and Cottyn, “La misère des Flandres”.

19. Van Isacker, “Mijn land in de kering,” 68–70.

20. Deneckere, *Sire, het volk mort!*, 77–129.

21. Van Ginderachter, “Arm Vlaanderen,” 184.

22. Ibid.

23. Lamberty, *De Vlaamse Opstanding*, 64–65.

24. Hanot, “De Brusselse tijdschriften,” 107–108.

25. Ronse, “Het arme kind,” 123–124.

26. Eekma, “De zusters,” 208–212.

27. Duvilers, “Pleitrede voor den armen,” 118, 120.

28. See *Vlaemsche bloemkorf*, 322–334.

29. Kelleher, *The Feminization of Famine*; Corporaal, Cusack, Jansen (eds.), *Recollecting Hunger*.

30. See also Absallis, *Het slechte geweten van Vlaanderen*, 65.

31. Another response came from Snellaert, *Wael en Vlaming*, in which he used the expression “the misery of Flanders” (“het ongeluk van Vlaenderen”), 4.

32. Sleeckx, “Vlaemsche zelfopoffering (1846–1847),” 4.

33. See Van den Berg and Couttenier, *Alles is taal geworden*, 374–82.

34. De Vlaemsche stem, vol. 1 (1846), 285–8.

35. Jan van Beers, “Bij de wiege van een kind des armen,” 44–48.

36. Van Kerckhoven, “Vrouw Lisa,” 298–302.

37. Conscience, “Redevoering,” 65: “Vlaanderen! Vlaanderen! Nu is die naam een gebed, – eene klacht die oprijst uit een onmeetbaar graf- een doodsschreeuw die vergaat op de lippen der stervenden”.

38. Conscience, “Redevoering,” 65: “het rijk des hongersnoods”.

39. “En misschien – de geest der toekomst roept het aan mijn oor – misschien zal het schoone Vlaanderen nog eens het hoofd uit het graf verheffen, en aan den hemel der natiën blinken als de star der nijverheid en der volkskracht!.” Conscience, “Redevoering,” 70.

40. *Het Handelsblad* (10 maart 1847).

41. Conscience, *Een goed hart*, 4.

42. Ibid., 9.

43. “Hongersnood in België,” in Dautzenberg and Van Duyse, Verhalen uit de geschiedenis van België, 31.34. The book was reprinted in 1862 and 1866.

44. “De Wil, die onderwerping vraagt,/Zal troosten, heelen na de slagen; ‒ Moed! Stervling, moed! Om ’t lot te dragen”. Van Ackere-Doolaeghe, “De aardappelplaag,” 50.

45. On the use of the expression “Poor Flanders” throughout the nineteenth century, see Dekkers, “De economische achterstand van Vlaanderen”.

46. Van Isacker, *Mijn land in de kering*, 67–73; Wils, *De politieke orientering van de Vlaamse beweging*, 31–36; Deneckere, *Sire, het volk mort*, 77–129; Vanginderachter, “Arm Vlaanderen,” 184; and Absallis, *Het slechte geweten van Vlaanderen*, 64–66, 111, 178.

47. See the editions of the work of Conscience on the digital platform *Digitale Bibliotheek der Nederlandse letteren* (www.dbnl.org).

48. Van Hemeldonck, *Hongersnood in Vlaanderen*, 27–28.

49. Bruneel, Beeckaert and Cottyn, “La misère des Flandres” (also available in French on. https://www.les-plats-pays.com/article/la-misere-des-flandres-dans-la-perspective-transatlantique.), *De Canon van Vlaanderen*, 178–181 and online: https://www.canonvanvlaanderen.be/events/de-aardappelcrisis/. Also see the informative websites of the Centre of Agricultural History (CAG): https://cagnet.be/page/landbouw-1750–1880 crisis.

50. For the food riots, see Van der Wiel, “Brood, brood, hongersnood!” and Paping and Tassenaar, “The Consequences of the Potato Disease,” 165–67.

51. Beets, *Troost der armen.*

52. These prices are mentioned in *Opregte Haerlemsche Courant* (23/12/1845).

53. Schotel, *Tollens en zijn tijd*, 259–60.

54. Van Koetsveld, “Klaas de landverhuizer”.

55. *Algemeen Handelsblad*, 24 January 1846.

56. Heldring, *Leven & arbeid*, vol. 2, 128.

57. See on the cultural responses to these and other floods Jensen, *Water: A Dutch Cultural History* and to the cholera outbreaks Jensen, “Cultural Resilience”.

58. This novel is also discusses in Corporaal and Jensen, “Famine and ‘Hongersnood’ as Transnational Memory”.

59. Within the project Heritages of Hunger (https://www.ru.nl/en/research/research-projects/heritages-of-hunger), funded by NWO, several activities have been undertaken to raise awareness of the nineteenth-century famines in the Low Countries. We, for instance, developed a series of lessons for primary schools, in cooperation with the Wetenschapsknooppunt Radboud Universiteit (WKRU). Furthermore, we compiled a database with primary sources from several European countries, which will be launched in 2025. A final example is the inclusion of the potato blight in the historical overview of disasters in the Netherlands: Jensen, *Rampen*, 174–180.

60. Orjuela, “Remembering/forgetting hunger”.

61. Mark-FitzGerald, *Commemorating the Irish Famine*, 61.

62. Jensen, *Water: A Dutch Cultural History.*

63. The Saint-Elisabeth’s Flood was commemorated with many events in Dordrecht and its surroundings. Preparations for the bicentennial commemoration of the Storm Flood of 1825 are already in full swing in several villages and cities around the Ijsselmeer (back then: Zuiderzee).

64. Heldring, *Wat te denken van en wat te doen in den aardappelennood?*, 29.

65. For an impression of the diversity in reactions and controversies, see the series on *de lage landen*: https://www.de-lage-landen.com/series/canon-en-co (Accessed January 9, 2024).

66. Buelens, “Misleidend, subversief of geniaal”.
